# Exploring Fibrosis Pathophysiology in Lean and Obese Metabolic-Associated Fatty Liver Disease: An In-Depth Comparison

**DOI:** 10.3390/ijms25137405

**Published:** 2024-07-05

**Authors:** Milena Vesković, Milka Pejović, Nikola Šutulović, Dragan Hrnčić, Aleksandra Rašić-Marković, Olivera Stanojlović, Dušan Mladenović

**Affiliations:** 1 Institute of Pathophysiology, Faculty of Medicine, University of Belgrade, Dr Subotića 9, 11000 Belgrade, Serbia; 2 Primary Health Center “Vračar”, Velimira Bate Živojinovića 16, 11000 Belgrade, Serbia; 3 Institute of Medical Physiology, Faculty of Medicine, University of Belgrade, Višegradska 26, 11000 Belgrade, Serbia

**Keywords:** lean NAFLD, fibrosis, animal models, MCD diet, CCl_4_

## Abstract

While obesity-related nonalcoholic fatty liver disease (NAFLD) is linked with metabolic dysfunctions such as insulin resistance and adipose tissue inflammation, lean NAFLD more often progresses to liver fibrosis even in the absence of metabolic syndrome. This review aims to summarize the current knowledge regarding the mechanisms of liver fibrosis in lean NAFLD. The most commonly used lean NAFLD models include a methionine/choline-deficient (MCD) diet, a high-fat diet with carbon tetrachloride (CCl_4_), and a high-fructose and high-cholesterol diet. The major pro-fibrogenic mechanisms in lean NAFLD models include increased activation of the extracellular signal-regulated kinase (ERK) pathway, elevated expression of α-smooth muscle actin (α-SMA), collagen type I, and TGF-β, and modulation of fibrogenic markers such as tenascin-X and metalloproteinase inhibitors. Additionally, activation of macrophage signaling pathways promoting hepatic stellate cell (HSC) activation further contributes to fibrosis development. Animal models cannot cover all clinical features that are evident in patients with lean or obese NAFLD, implicating the need for novel models, as well as for deeper comparisons of clinical and experimental studies. Having in mind the prevalence of fibrosis in lean NAFLD patients, by addressing specific pathways, clinical studies can reveal new targeted therapies along with novel biomarkers for early detection and enhancement of clinical management for lean NAFLD patients.

## 1. Introduction

MAFLD is presented as a disease that includes diffuse infiltration of hepatic tissue with fat, including the deposition of fat, which is non-inflammatory; liver fibrosis; and nonalcoholic steatohepatitis, which can lead to liver cirrhosis, liver failure, and cancer [1,2]. The two-hit hypothesis implies that the ‘first-hit’—steatosis—increases the vulnerability of the liver to injury, mediated by inflammatory cytokines/adipokines and oxidative stress, which represent ‘second-hits’ leading to steatohepatitis and fibrosis [3]. Nowadays, the “two-hit” hypothesis is considered obsolete, and the new hypothesis that found its place in the clarification of pathogenetic mechanisms is called the “multiple-hit”. The “multiple-hit” hypothesis describes multiple insults that act together, such as insulin resistance, nutritional factors, gut microbiota, adipose tissue hormones, and a variety of genetic and epigenetic factors [4]. With the emergence of new theories, new terminology is also introduced, so the latest term for steatotic liver disease associated with metabolic syndrome is metabolic dysfunction-associated steatotic liver disease (MASLD). MASLD is now considered the main cause of liver-related morbidity and mortality [5].

In an article published in 2022 by Jiaye Liu et al., scientists identified 116 relevant studies with more than two and a half million participants in the general population with an estimated prevalence of metabolic dysfunction-associated fatty liver disease of 50.7% (95% CI 46.9–54.4) in a group of obese adults, with the prevalence significantly higher amongst males. The conclusion was that MAFLD has an astoundingly high prevalence rate in obese and overweight patients [6]. Studies also show that the most frequent liver disease in the Western world with a prevalence of 20% is nonalcoholic fatty liver disease (NAFLD) [7]. It can be said that metabolic-associated fatty liver disease (MAFLD) is also the disease of the new age considering the fact that the prevalence is rapidly increasing globally due to sedentary lifestyles, poor nutrition, obesity, urbanization, and healthcare accessibility. However, in obese patients, NAFLD is primarily characterized by metabolic dysfunction, which includes excessive fat accumulation, high body mass index, insulin resistance, chronic inflammation, and dyslipidemia. On the other hand, lean NAFLD is not linked with these metabolic disturbances. Instead, it might originate from genetic factors, gut microbiota alterations, and hormonal changes. This divergence in underlying causes suggests that the mechanisms involved in liver damage and disease progression in lean NAFLD might be different, often resulting in more pronounced fibrosis [8].

In the analysis of metabolic dysfunction-associated steatohepatitis, the progression and development of hepatocellular carcinoma (HCC) liver fibrosis represent important predictors [9]. The common problem with liver fibrosis in patients with NAFLD is its delayed diagnosis because this type of disease is often overlooked. Stage F3 of liver fibrosis and stage F4 of cirrhosis are the most precise predictors of increased mortality not only in liver-related diseases but also in diseases of cardiovascular etiology. These patients are at great risk of developing hepatocellular carcinoma [7].

Generally, in obese individuals presenting with a constellation of metabolic syndrome components, diagnosis making is quite straightforward. In contrast, lean NAFLD can be more challenging since patients do not exhibit typical risk factors such as obesity and high body mass index (BMI), which consequently require different therapeutic approaches. Therapeutic strategies in obese NAFLD patients are focused on weight loss, improving insulin sensitivity, and reducing inflammation. However, these interventions may not be effective in the same way in lean NAFLD individuals. So far, physical activity, healthier dietary patterns, and sleep interventions have beneficial impacts on lean NAFLD with strict management of comorbidities such as diabetes mellitus type 2, dyslipidemia, and hypertension [10]. Recent recommendations suggest that all patients with lean NAFLD should undergo risk stratification for hepatic fibrosis and a determination of those with a higher risk for disease progression [11].

The aim of this review is to comprehensively examine the pathophysiological mechanisms of liver fibrosis in lean and obese individuals with MASLD by reviewing recent literature regarding research using lean NAFLD models.

## 2. MAFLD Pathogenesis

**Figure 1:**
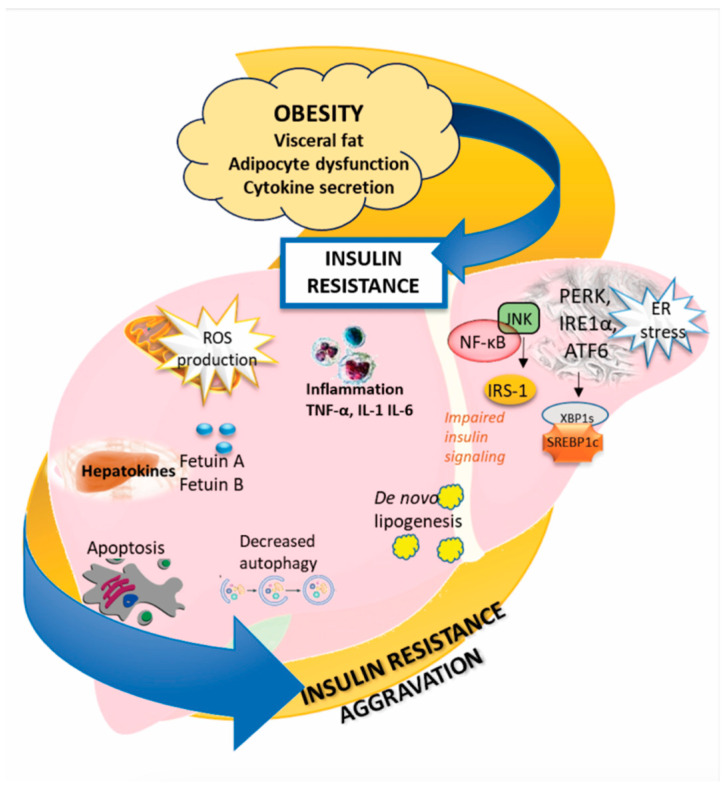
Insulin resistance in MAFLD pathogenesis. In obesity, dysfunctional adipocytes increase circulating free fatty acids, which are taken up by the liver, leading to triglyceride accumulation and exacerbation of hepatic insulin resistance. Hepatic insulin resistance triggers liver inflammation through proinflammatory cytokines, oxidative stress due to excess reactive oxygen species, and ER stress from protein misfolding, all of which disrupt cellular functions. These stress responses, along with apoptosis and reduced autophagy, cause hepatocytes to release hepatokines, further aggravating systemic insulin resistance and creating a feedback loop that worsens liver dysfunction.

Obesity represents a significant risk factor for metabolic disorders, primarily due to the dysfunction of adipocytes, which play a crucial role in lipid storage and endocrine function. In obese individuals, adipocytes become hypertrophic and dysfunctional, leading to altered secretion of adipokines such as leptin and adiponectin. Obesity, distinguished by the anomalous accumulation of body fat, is a risk factor or an aggravating factor of MAFLD as it is also a risk factor for type II diabetes. Adipose tissue dysfunction contributes to systemic insulin resistance by promoting chronic low-grade inflammation and ectopic lipid accumulation [12]. Insulin resistance in obesity is characterized by impaired insulin signaling pathways, particularly in adipose and hepatic tissue. Physiologically, insulin regulates glucose levels, lipolysis of adipose tissue, and hepatic synthesis of very low-density lipoprotein (VLDL). In patients with insulin resistance, there is an increased release of adipose tissue free fatty acids (FFAs) accompanied by increased hepatic uptake of FFAs, which are then transformed into hepatic triglycerides, resulting in hepatic steatosis [13]. On a cellular level, an increased concentration of fatty acids induces endoplasmic reticulum (ER) stress, obstructing the secretion of apolipoprotein B100 and contributing to the progression of steatosis [14]. There are also findings that suggest that hepatic steatosis may be the start-off event causing insulin resistance in lean individuals [15]. Discussing dyslipidemia in MAFLD, carbohydrate regulatory element binding protein (ChREBP) is activated by products of glucose metabolism after insulin and the liver X receptor activate SREBP1c (sterol regulatory element binding protein 1c). This activation cascade continues by increasing the expression of genes related to lipogenesis [16]. Former studies have shown that the stimulation of the hepatic mitochondrial capacity by the larger availability of lipids in the obese MAFLD patients’ liver eventually leads to exaggerated oxidative stress and decreased mitochondrial efficacy, contributing to the progression of steatohepatitis [17]. Metabolic derangement in the liver triggers a cascade of deleterious processes, including inflammation, oxidative stress, and endoplasmic reticulum (ER) stress. Inflammation in the liver is mediated by proinflammatory cytokines such as tumor necrosis factor (TNF)-α, interleukin (IL)-1, and IL-6, which are upregulated in the context of adipocyte dysfunction and insulin resistance. Oxidative stress results from an imbalance between reactive oxygen species (ROS) production and antioxidant defenses, leading to cellular damage [1,18]. Concurrently, ER stress, caused by the accumulation of misfolded proteins, activates the unfolded protein response, further disrupting insulin signaling. During stress conditions, misfolded or unfolded proteins accumulate in the ER, triggering the unfolded protein response (UPR). The UPR is activated in order to restore normal cell function, the degradation of misfolded proteins, and the production of more chaperones that are responsible for protein folding. Three types of ER-resident stress sensors are activated and include inositol-requiring enzyme (IRE1), PKR-like ER kinase (PERK), and activating transcription factor 6 (ATF6) [19]. UPR is involved in the regulation of lipid homeostasis in hepatocytes, suggesting that prolonged ER stress may contribute to MASLD pathogenesis by affecting lipid metabolism in hepatocytes through altered VLDL secretion, inducing de novo lipogenesis, impaired insulin signaling, and autophagy. Additionally, stressed hepatocytes release hepatokines such as Fetuin A and B and Selenoprotein P, which are signaling molecules that exacerbate systemic insulin resistance and inflammation. This feedback loop between liver dysfunction and systemic metabolic disturbances perpetuates and worsens hepatic insulin resistance, creating a vicious cycle of metabolic dysregulation that is central to the pathology of obesity-related liver diseases (Figure 1) [20,21].

The following are some abbreviations used: ROS—reactive oxygen species, TNF—tumor necrosis factor, IL—interleukin, IRE1—inositol-requiring enzyme, PERK—PKR-like ER kinase, ATF6—activating transcription factor 6, ER—endoplasmic reticulum, XBP1—X-box binding protein 1, SREBP—sterol regulatory element binding protein, JNK—c-jun-N-terminal kinase, IRS1—insulin receptor substrate-1, and NF-kB—nuclear factor kappa B.

Interconnection between the gut and the liver plays an important role in the development and progression of MAFLD. The gut microbiota and their metabolites are an important segment of the “multi-hit” hypothesis since bile acids and short-chain fatty acids (SCFAs) are involved in the regulation of lipid metabolism in the liver and inflammation in MAFLD [22].

## 3. Gut–Liver Axis and the Role of the Microbiome in Liver Disease Development

The biology of the gut–liver communication axis has been explored in detail in the past years. This mechanism is an important part of pathogenesis in liver disease as in many other types of diseases. Of course, the complexity of the microbiome has to be elucidated when we talk about the significance of the gut–liver communication axis. There are already some clinical studies conducted with the intention of proving some key elements in the interaction between the gut and the liver [23,24,25].

Probably the most important molecules in this interaction are bile acids (BAs), which help with the absorption of dietary fats, cholesterol, and fat-soluble vitamins.

The greater part of the BAs is reabsorbed and transported back to the liver while the remaining BAs are metabolized by the intestinal microbiota and transformed into secondary BAs, which are transported to the liver via portal circulation [26,27]. There is an unbreakable bond between BAs and the gut microbiota in a way that they adjust to each other. BAs are in control of the production of angogenin 1 and RNAse family member 4, antimicrobial peptides that affect gut microbial overgrowth and, eventually, its dysfunction [28,29]. When the balance between primary and secondary BAs is disturbed, metabolic stress develops [30,31,32,33]. Consequently, intestinal permeability is damaged due to gut inflammation and dysbiosis. That way, microbes and their molecules can reach the liver and cause inflammation and injury. The specific bacteria, Akkermansia muciniphila, precisely its reduced quantity, is found to be responsible for the thinning of the mucus layer, escalating inflammation and encouraging liver damage [34,35]. Microbial dysbiosis of the lower gastrointestinal tract is connected to liver damage due to MAMPs’ increased translocation and their causing of localized inflammation through receptors on Kupfer cells [36] and hepatic stellate cells [37,38]. Several studies have shown the important role of the gut microbiota in NAFLD [39]. Boursier et al. [40] found that the bacterial genera Prevotella was reduced and Bacteroides and Ruminococcus were notably increased in NASH patients with stage 2 fibrosis or higher. Loomba et al. [23] discovered an increased plethora of Escherichia coli and Bacteroides vulgatus in advanced fibrosis patients. These studies demonstrate the connection between Gram-negative bacteria and the progression of liver fibrosis [41].

Also, considering the fact that NAFLD represents the hepatic emblem of metabolic syndrome, a study was conducted amongst patients with coeliac disease (CD) with the aim of evaluating the prevalence of NAFLD in CD patients treated with a gluten-free diet (GFD). The conclusion that was reached was that more than one-third of patients with CD adhering to a GFD had parallel NAFLD, resulting in a threefold higher risk compared to the general population [42].

## 4. Liver Fibrosis Pathogenesis

Liver fibrosis occurs as a result of chronic liver injury and often results in the development of cirrhosis and HCC [43]. Fibrosis is defined by excessive accumulation of the extracellular matrix (ECM) and decreased degradation as a response to chronic injury, which can lead to permanent scarring, organ malfunction, and, ultimately, end-stage liver disease. The most common causes of liver fibrosis are viral hepatitis, alcoholic steatohepatitis, and nonalcoholic steatohepatitis. Other causes of liver fibrosis include autoimmune hepatitis, cholestasis, metabolic disorders, primary biliary cirrhosis, and drugs and toxins [44,45]. During liver injury, endogenous triggers are released from damaged cells and facilitate inflammation [46], which is mediated primarily by macrophages, Kupffer cells, dendritic cells, and mast cells. These inflammatory cells release profibrotic markers that stimulate the synthesis of ECM proteins and inhibit the degradation of ECM proteins [47]. Fibrotic tissue accumulates in the portal area and pericentral and perisinusoidal spaces, with the tendency to form bridging structures and nodules in cirrhosis [48]. Loss of normal tissue architecture followed by impaired function leads to liver insufficiency, ultimately leading to organ transplantation as the only solution.

In the pathogenesis of liver fibrosis, both parenchymal and nonparenchymal cells are involved. Activation of myofibroblasts that produce ECM proteins plays a critical role in the pathogenesis of liver fibrosis. The most important source of myofibroblasts, and therefore ECM proteins, are activated hepatic stellate cells (HSCs). The ECM is a complex web of proteins that contain different types of collagens and its composition changes in fibrosis. It has an important role in the proliferation and differentiation of cells, cell survival, and likely metabolic pathways. ECM configuration in nonalcoholic steatohepatitis (NASH) has yet to be uncovered [49]. The ECM, among collagen, consists of many noncollagenous glycoproteins, glycosaminoglycans, and proteoglycans. Given that the amount of these proteins is severely changed in fibrosis, the configuration of the ECM is altered. The most powerful cytokine in fibrogenesis is transforming growth factor-β (TGF-β), a triggering factor in HSC stimulation and liver fibrosis [50]. Merged inhibition of TGF-β and IL-13 signaling diminishes fibrotic mechanisms more suitably than TGF-β alone. Another factor, equally important in liver fibrosis, is platelet-derived growth factor (PDGF). Other HSC factors include Indian hedgehog, sonic hedgehog, and osteopontin [51]. Immune cells, such as natural killer (NK) cells, B cells, and NKT cells, among the crucial hepatocyte–macrophage–HSC network participate in the promotion of liver fibrosis [52].

Hepatocytes also participate in the activation of HSC via numerous mechanisms. The main one implies inflammation caused by stress and the death of cells, which is followed by macrophage sourcing and the secretion of mediators such as TGF-β, thus activating the hepatocyte–macrophage–HSC system as a principal fibrogenic response in NASH. HSCs can also be activated directly by profibrogenic mediators like Hh ligands, apoptotic bodies, or the previously mentioned osteopontin [53]. Many reports demonstrate HSC activation after the cells’ absorption of apoptotic bodies both in vitro and in vivo, proposing direct connections between the death of hepatocytes and the activation of HSC [54]. A characteristic feature of NASH, hepatocyte ballooning, is related to a higher risk for fibrosis development. This form of cells, considered ‘neither dead nor alive’, stimulates HSC activation by means of the aforementioned sonic hedgehog (SHH) [51].

Hepatocyte TAZ represents a paralogue of YAP and a main, irreplaceable component of the HIPPO-YAP/TAZ-TEAD signaling cascade. TAZ upregulation was not to be found in simple steatosis, which implies that it could be involved in the transformation from simple steatosis to NASH. The suppression of TAZ could lead to a decrease in the expression of its main goal Indian Hedgehog (IHH), which all consequently suppress NASH-induced fibrosis and decrease hepatocyte death and inflammation [55]. Notch activity is considerably boosted in murine and human NASH, and it represents a pathway in the differentiation of hepatocyte progenitors in favor of cholangiocytes [56]. In addition, noteworthy is that Notch activation is followed by glucose intolerance because it stimulates FoxO1 activation at gluconeogenic promoters, which can give us insight into the interrelation between type 2 diabetes and hastened NASH development.

Another activator of the hedgehog pathway is ER stress, which is a frequent occurrence in NASH. ER stress influences the expansion of expressed SHH, an Hh ligand. Findings advocate that ballooned hepatocytes create Hh ligands which then have paracrine profibrogenic traits. In addition, studies show a dual role of SHH in NAFLD minimizing accumulation of triglycerides in the liver while encouraging the development of fibrosis [49]. There can be an interaction between the YAP/TAZ and Notch pathway; however, those connections remain to be explored. Fibrogenesis without changing liver injury is promoted by Notch, and TAZ influences both processes. Today, we know that the majority of patients with steatosis do not develop NASH. Increasing the concentration of lipids could cause the stimulation of NASH-promoting pathways such as TAZ, Notch, or Hh. It is yet unknown whether steatosis is only a required element that makes the liver sensitive to injury. Studies have shown that peroxidized lipids and saturated fatty acids instigate a proinflammatory macrophage phenotype [57]. HSCs are activated and fibrosis is encouraged through increased expression of Toll-like receptor (TLR)-4 in high-cholesterol-diet mice, so it appears that cholesterol influences changes in not only hepatocytes but also HSCs and macrophages [58].

TGF-β is the most powerful mediator of HSC activation and fibrosis and is reinforced in macrophages. Scar-associated macrophages (SAMs) express genes that promote fibrosis, including IL-1β, SPP1, PDGFB, and TNFSF12, and HSCs/fibroblasts contain appropriate receptors, making the SMA-HSC/fibroblast axis a necessary contributor to the evolution of fibrosis [59]. IL-1β derived from macrophages increases the concentration of ECM-producing myofibroblasts. Osteopontin, which is encoded by SPP1, directly encourages HSC activation, and the proliferation of said cells is stimulated through TNFSF12 and PDGFB. Besides these functions, liver macrophages also influence the degradation of ECM during the regression stage. Also, one of the many important roles of liver macrophages is inflammation regulation in NASH. They express elevated levels of TLR4, which then results in increased production of TNF and IL-1β and inflammation stimulation in NAFLD [57]. The control of liver inflammation, fat accumulation, hepatocyte death, and fibrosis by cytokines originating from macrophages are probably closely interconnected and collaborate to propel the advancement of NASH.

## 5. Lean versus Obese MAFLD

Lean NAFLD presents a unique set of characteristics compared to typical NAFLD cases. It primarily affects individuals with a lower body mass index (BMI), often those who are not obese or overweight. Despite their lean appearance, these individuals exhibit signs of liver fat accumulation, inflammation, and potential progression to more severe liver conditions like fibrosis and cirrhosis. Lean NAFLD is indeed associated with metabolic abnormalities, despite the individual’s lower BMI. These abnormalities can include insulin resistance, dyslipidemia, and other metabolic disturbances [60]. Compared to obese NAFLD individuals, lean NAFLD individuals may have a lower prevalence of these metabolic disorders, but they are still significant contributors to the disease process. The metabolic abnormalities in lean NAFLD individuals contribute to liver fat accumulation, inflammation, and the progression of the disease. When comparing lean and obese NAFLD, major points that should be considered include body composition, metabolic profile, disease severity, molecular mechanisms, and clinical management [61].

### 5.1. Body Composition

Lean NAFLD presents a unique clinical phenotype characterized by liver fat accumulation in individuals with a lower BMI and less overall adiposity compared to obese NAFLD counterparts [62]. Understanding the differences in body composition between lean and obese NAFLD individuals is crucial for elucidating symptomatology, clinical presentation, and management strategies for this complex liver condition. Lean NAFLD individuals typically have a BMI within the normal range, often below 25 kg/m^2^, indicating a lower amount of total body fat [63]. Despite their lean appearance, these individuals may exhibit a distinct pattern of fat distribution characterized by visceral adiposity or ectopic fat deposition, particularly within the liver. While obesity is a well-established risk factor for NAFLD, lean individuals with NAFLD demonstrate that adiposity alone does not fully account for the development of liver fat accumulation and associated metabolic abnormalities. One key aspect of body composition differences between lean and obese NAFLD individuals lies in the distribution of adipose tissue [64]. In obese individuals, excess adiposity is typically distributed subcutaneously and viscerally, contributing to a higher BMI and increased risk of metabolic disorders such as insulin resistance and dyslipidemia. In contrast, lean NAFLD individuals may have less subcutaneous and visceral adipose tissue but still exhibit ectopic fat deposition within the liver, suggesting a unique metabolic phenotype that predisposes them to liver fat accumulation despite their lower BMI [61,65]. The most important risk factors associated with lean NAFLD are high triglyceride levels and changes in waist circumference [66,67]. Recent studies showed that lean individuals with larger waist circumferences have a higher risk of diabetes, atherosclerosis, hypertension, and liver fibrosis [68,69], and liver biopsies from lean NAFLD patients showed a higher rate of inflammation and ballooning of hepatocytes compared to the obese NAFLD group [70].

### 5.2. Disease Severity

Disease severity in NAFLD varies between lean and obese individuals, reflecting differences in body composition, metabolic profile, and underlying molecular mechanisms. Lean NAFLD individuals, despite their BMI and reduced adiposity, are not immune to disease progression. While they may have a lower prevalence of metabolic comorbidities such as insulin resistance and dyslipidemia compared to obese NAFLD individuals, lean NAFLD patients can still experience significant liver damage and complications. Studies have shown that lean NAFLD individuals may exhibit more severe liver histology at presentation, including higher rates of liver inflammation and fibrosis, suggesting that metabolic abnormalities and other factors play a prominent role in driving disease progression. A recent clinical cohort study that included over 130,000 subjects confirmed that NAFLD in lean subjects is more severe for fibrosis, the progression of liver disease, chronic kidney disease, and overall mortality [71]. A similar study among Indian patients that included around 1000 patients with NAFLD showed similar disease severity among lean and obese NAFLD individuals even though lean patients exhibited fewer metabolic risk factors [72].

### 5.3. Pathophysiology

Obese NAFLD individuals present with a higher burden of metabolic disorders and are at increased risk of developing advanced liver disease. Excess adiposity contributes to a proinflammatory and insulin-resistant state, promoting lipid accumulation within hepatocytes and exacerbating liver injury [73]. Obese NAFLD patients are more likely to have features of metabolic syndrome, such as central obesity, hypertension, and dyslipidemia, which further exacerbate liver damage and increase the risk of cardiovascular complications. Histologically, obese NAFLD individuals may exhibit a spectrum of liver pathology ranging from simple steatosis to NASH, characterized by hepatocyte injury, inflammation, and fibrosis. The presence and severity of fibrosis are important predictors of long-term outcomes in NAFLD, with advanced fibrosis indicating an increased risk of liver-related morbidity and mortality [74]. Obese NAFLD patients are also at higher risk of developing cirrhosis, HCC, and liver-related mortality compared to lean NAFLD individuals.

In lean NAFLD individuals, the mechanisms driving liver fat accumulation may differ from those with obesity. Genetic predisposition, altered lipid metabolism, mitochondrial dysfunction, and insulin resistance independent of adiposity have been proposed as potential contributors to lean NAFLD pathogenesis. Despite their lower BMI, lean individuals with NAFLD may still exhibit metabolic abnormalities such as insulin resistance and dyslipidemia, albeit to a lesser extent than their obese counterparts [67]. These metabolic disturbances contribute to liver fat accumulation and inflammation, further highlighting the complex interplay between genetics, lifestyle factors, and metabolic health in NAFLD pathogenesis. Clinically, lean NAFLD individuals may present with similar features as obese NAFLD patients, including elevated liver enzymes, hepatic steatosis on imaging studies, and histological evidence of liver inflammation and fibrosis. However, lean NAFLD individuals may be at a lower risk of metabolic comorbidities such as type 2 diabetes and cardiovascular disease compared to obese NAFLD individuals [61]. Nonetheless, both lean and obese NAFLD individuals are at increased risk of progression to advanced liver disease, including fibrosis, cirrhosis, and hepatocellular carcinoma, underscoring the importance of early detection and intervention [71].

In lean NAFLD individuals, adipose tissue dysfunction exerts higher adverse adipokine and cytokine profiles, ultimately causing low-grade inflammation, also known as metaflammation. Alteration in gut microbiota also contributes to NAFLD progression and is tightly connected to diets enriched in cholesterol [75]. Studies using a cholesterol-rich diet showed the development of NAFLD followed by weight loss and significant systemic inflammation. Similarly, a methionine-choline-deficient (MCD) diet is used to induce NAFLD in mice followed by weight loss and dyslipidemia. An MCD diet caused total cholesterol, high-density lipoprotein (HDL), and triglyceride levels to progressively decrease, whereas low-density lipoprotein (LDL) levels progressively increased. Focal fatty change in the liver appeared after 2 weeks, whereas diffuse fatty change with severe inflammation and ballooned hepatocytes were evident after 6 weeks [76]. However, the same model of lean NAFLD in mice confirmed the presence of liver inflammation evident through increased proinflammatory cytokines IL-6 and TNF-α with decreased anti-inflammatory IL-10 [77]. Changes in lipid metabolism and the presence of hepatic and systemic inflammation may be the major contributing factors involved in lean NAFLD pathogenesis. A recent study by Huang et al. [76] pointed to the potential role of macrophage sterol regulatory element binding protein (SREBP) cleavage-activating protein (SCAP) in amplifying the inflammatory response in adipose and liver tissues, leading to metabolic disorders participating in the development of lean NAFLD with metabolic changes. The proposed mechanism involves the inflammatory response after SCAP-mediated activation of the stimulator of the interferon gene (STING)–NF-κB signaling pathway in macrophages. SCAP is a cholesterol sensor that regulates signal transduction for intracellular cholesterol homeostasis, and its dysregulation may affect the development of metabolic disorders, suggesting that cholesterol metabolism plays an important role in lean NAFLD pathogenesis [75,78]. The dietary inflammatory index was shown to be positively correlated to both obese and lean NAFLD and liver fibrosis in the general population. However, the impact of a proinflammatory diet was less prominent in lean individuals compared to obese ones [79].

## 6. Animal Models of Lean NAFLD

Numerous animal models are used for NAFLD investigation, and the best model should include all metabolic abnormalities, serum lipid profile alteration, insulin resistance, liver fat accumulation, and obesity. However, there are animal models that are used for the development of lean NAFLD, which include fatty liver change but without obesity and subcutaneous fat accumulation [80]. These models include dietary, genetic, toxic, and combined factors.

The mostly used dietary models are the MCD diet [76], choline-deficient L-amino-defined diet (CDAA) [81], choline deficiency, L-amino acid-defined high-fat diet (CDAA+HF) [82], high-fructose diet [83], and cholesterol-rich diet [84]. Genetic models used for lean NAFLD development commonly used are knockout strains lacking PPAR-α or CD36. Tetracycline and carbon-tetrachloride (CCl_4_) can be used as a toxic model, while CCl_4_ with a high-fat diet (HFD) is used as a combined model for NASH-induced liver fibrosis [80] (Table 1).

## 7. Mechanisms of Liver Fibrosis in Different Models of Lean NAFLD

The methionine- and choline-deficient (MCD) diet is a widely used animal model to study the pathogenesis of NAFLD/NASH. The MCD diet is characterized by its lack of methionine and choline, two essential nutrients required for hepatic lipid metabolism. Methionine is an essential amino acid necessary for the synthesis of glutathione, an important antioxidant, while choline is crucial for the production of very-low-density lipoprotein (VLDL), which exports triglycerides from the liver. Typically, an MCD diet composition includes a high content of carbohydrates (40–70%), moderate to high fat content (10–40%), and a moderate amount of proteins (10–20%), with a lack of methionine and choline [76,77].

The duration of MCD diet feeding in animal studies varies, typically ranging from 2 to 24 weeks. Shorter durations (2–6 weeks) are often used to induce simple steatosis, while longer durations (8–24 weeks) are used to develop more severe liver damage, including inflammation and fibrosis. Animals fed with an MCD diet develop several key features of NAFLD/NASH, which include hepatic steatosis, presented particularly as macrovesicular fsteatosis; hepatic inflammation characterized by increased levels of inflammatory cytokines and infiltration of inflammatory cells; and oxidative stress due to the depletion of glutathione and other antioxidants. Prolonged MCD feeding leads to fibrosis, characterized by increased expression of profibrogenic factors [76,77,78]. Machado et al. [85] conducted a study comparing MCD diet- and Western diet-induced NAFLD in mice for 8 and 16 weeks, respectively, to develop both lean and obese NAFLD models. Results showed increased expression in α-smooth muscle actin (SMA), collagen type I, and TGF-β in both lean and obese animals when compared to the control group. However, there was a statistically significant difference between these two experimental groups suggesting that lean NAFLD has more pronounced profibrogenic potential than the obese NAFLD model. Similarly, a study by Zhen et al. [86] showed that the MCD diet induced liver fibrosis evident on Masson’s trichrome staining. Profibrogenic effects were mediated through an increase in the activation of extracellular signal-regulated kinase (ERK), which is a key component of the MAPK signaling pathway. In the MCD diet-fed group, the expression and protein levels of α-SMA, collagen type I, fibronectin, and TGF-β were significantly increased, contributing to liver fibrosis development [86]. In MCD diet-induced steatohepatitis, Leclere et al. [87]. showed that the fibrosis priming markers TGF-β1, collagen type 1, and tissue inhibitor of metalloproteinase (TIMP)-1 were upregulated upon administration of the MCD diet [87,88]. This indicates that the lean model of NAFLD induced by MCD diet fibrosis is developed by an increase in profibrogenic factors and metalloproteinase inhibitors. An additional mechanism by which the MCD diet induces liver fibrosis is through an increase in tenascin-X (Tnxb), a large extracellular matrix glycoprotein, and its augmented expression is caused by the decreased expression of miR-378a-5p and miR-486-5p. This finding was accompanied by increased collagen type 1, α-SMA, and TGF-β expression in the liver [89]. Insulin-like growth factor binding proteins (IGFBP)-1, -2, and -7, through their consequent senescence, have a role in the progression of NAFLD and its associated fibrosis, being a plausible determinant in the progression from steatosis to NASH in the MCD diet mice model [90]. In the study by Wang et al., it has been shown that X-box binding protein (XBP)-1 controls macrophage cGAS/STING/NLRP3 activation, representing a significant profibrogenic signaling pathway in MCD diet-induced and CCl_4_-induced lean NAFLD [91]. Using the MCD diet NAFLD murine model, Cao et al. [92] identified that the M2 macrophages promoted HSC autophagy by secreting prostaglandin E2 (PGE2) and binding its receptor EP4 on the surface of HSCs, which consequently enhanced HSC activation, extracellular matrix deposition, and liver fibrosis. These features are also evident in humans with NASH and represent the major similarities between animal models and human clinical presentation. However, animals on MCD diets often experience significant weight loss, resulting in a lean phenotype, whereas human NAFLD is often associated with obesity and metabolic syndrome. Animals on an MCD diet maintain or even improve insulin sensitivity, while humans with NAFLD often exhibit insulin resistance. An additional weakness of this animal model is that the lack of methionine and choline does not mimic typical human diets, which generally do not lack these nutrients [76,87,88,93] (Table 2).

These differences highlight the importance of using multiple models and approaches to study NAFLD to ensure a comprehensive understanding of the disease’s pathogenesis and potential treatments.

The choline-deficient, L-amino acid-defined (CDAA) diet is another commonly used animal model to study nonalcoholic fatty liver disease (NAFLD) and its progression to fibrosis. This model is particularly suitable for investigating the mechanisms underlying liver fibrosis in the context of NAFLD. The CDAA diet induces liver fibrosis relatively quickly (within weeks to a few months), enabling faster evaluation of potential therapeutic interventions [81,93]

This model reliably produces pericentral and perisinusoidal fibrosis, mimicking the fibrotic patterns observed in human NASH, making it a reliable tool for preclinical research. Similar to the MCD diet, the CDAA diet results in a lean phenotype in animals, which is useful for studying NAFLD independent of obesity and metabolic syndrome [81].

However, one of the significant limitations of the CDAA diet is the lack of metabolic syndrome features such as insulin resistance, hyperglycemia, dyslipidemia, and obesity, which are commonly associated with human NAFLD, affecting the translational potential of findings. Comparing MCD and CDAA diet models, animal body weight did not change in CDAA-fed animals. In addition, it has been shown that in both models, hydroxyproline content (a measure of collagen content per 100 mg of tissue) was increased, confirming fibrosis development. However, a CDAA diet enriched with 60% fat causes a more pronounced increase in hydroxyproline and histologically robust fibrosis. Total collagen content is increased in both models, especially after adding fat into a specific diet. Molecular analysis showed increased hepatic mRNA expression for TGF-β, α-SMA, collagen type I, and TIMP-1. This increase was evident after 8 weeks of the CDAA+HF diet. Severe fibrosis was found to be associated with the alteration of lipid metabolism-related genes that encode both isoforms of SCD1 and SCD2, which are important for de novo triglyceride synthesis [94]. SDCs catalyze a critical step in the production of active lipid-modified Wnt proteins and have been implicated in liver fibrosis [95,96]. A similar study using the CDAA+HF model confirmed fibrosis development evident through an increase in the expression of fibrosis-related genes TGF-β, α-SMA, and PDGF and also PPARγ, suggesting their participation in fibrosis pathogenesis [97]. In addition, gut barrier function was shown to play an important role in fibrogenesis, which was confirmed in a study by Enomoto et al. [98] demonstrating that liver fibrosis induced by the CDAA diet was ameliorated by rifaximin/lubiprostone treatment through repairment of gut barrier function. In the fibrotic model, there was increased translocation of lipopolysaccharides (LPS) to the liver and increased activation of toll-like receptor 4 signaling, which may be the potential mechanisms contributing to hepatic inflammation and progression to fibrosis. IL-8 was shown to play an important role in all stages of NASH induced by the CDAA+HF diet for 52 or 63 weeks, from initial inflammation to fibrosis progression and tumorigenesis [99].

The high-fructose diet has often been used for NAFLD induction. One of the primary advantages of the high-fructose diet model is its ability to induce features of metabolic syndrome, which makes the model highly relevant, as it mirrors the clinical presentation of NAFLD in individuals with obesity and metabolic syndrome. Additionally, the high-fructose diet is closely aligned with modern human dietary patterns, where fructose consumption, primarily through sugary beverages and processed foods, is prevalent [100].

Liver disease induced by a high-fructose diet ranges from simple steatosis to inflammation and fibrosis, mimicking the natural progression seen in humans. This progression allows researchers to study the different stages of NAFLD and identify potential intervention points [101]. Despite its advantages, one significant issue of this model is the variability in the severity of liver damage and the duration required to develop fibrosis [102]. This can make it challenging to use in studies with time constraints, as prolonged dietary exposure may be necessary to observe significant fibrosis. The high-fructose diet used for establishing the NASH model in mice was used in a study by Oliveira-Cordeiro et al. [103] to examine its effect on NASH progression to more severe liver diseases. In this animal model, collagen deposition in liver tissue was evident in the perivascular area. Fibrosis development in this model was at least partly mediated by increased mRNA expression for TGF-β, α-SMA, and collagen type 1. In addition, important activators of HSCs Smad3 and Yes1-associated transcriptional regulator (Yap-1) were also increased and involved in fibrogenesis and ECM deposition.

The high-cholesterol (HC) diet is another established animal model used to study NAFLD and its progression to fibrosis. This model focuses on the impact of high dietary cholesterol on liver pathology, offering a unique perspective on the disease’s development and underlying mechanisms [104].

Animals on an HC diet typically develop more severe liver pathology, including significant steatosis, inflammation, and fibrosis. This allows for the study of advanced stages of NAFLD and the mechanisms driving progression to NASH and fibrosis, making it valuable for investigating lipid metabolism and its dysregulation in NAFLD. The cholesterol-rich diet can be combined with other dietary models (e.g., high-fat or high-fructose diets) to study the synergistic effects of multiple dietary factors on NAFLD development and progression. Similar to previously mentioned dietary models, animals on a high-cholesterol diet may not fully exhibit the features of metabolic syndrome such as insulin resistance, hyperglycemia, and obesity, which are often present in human NAFLD patients. The duration required to observe significant fibrosis can vary, and the severity of effects may not always correlate with human NAFLD progression, potentially affecting the interpretation of findings [84,104,105]. A recent study with HC diet-induced NASH has provided new insights into the mechanisms linking HC diet uptake and liver fibrosis. The HC diet induces an accumulation of free cholesterol in HSCs, promoting TLR4 signal transduction by increasing membrane TLR4 levels. This consequently can activate HSC TGF-β signaling, which results in further activation of HCS and liver fibrosis [59]. Liver fibrosis in the high-fat high-cholesterol (HFHCD) model is mediated through an increase in gene expression for TGF-β1, αSMA, and collagen type I. During fibrogenesis, other genes involved in ECM remodeling are highly expressed in liver tissue, such as MMP2 and MMP9. In addition, a significant increase in TIMP 1, 2, and 3 expressions in liver tissue after 12 weeks of HFHCD has been confirmed, and their expression remained increased after returning animals to a standard diet even for 12 weeks, which suggests their role in slow fibrosis regression [106]. A similar novel model of NASH with advanced fibrosis is induced by HFHCD with supplementation with cholic acid. In 60% of rats with high cholic acid, severe fibrosis and cirrhosis develop. High expression of profibrotic markers TGF-β, collagen type I, and monocyte chemotactic protein-1 (Mcp-1) is reported in liver tissue and increased in a dose-dependent manner relative to cholic acid intake. Cholic acid together with cholesterol facilitates the development of NASH and plays an important role in the progression to liver fibrosis and cirrhosis [107].

Combining a high-fat diet with CCl_4_ administration is a robust animal model for studying NAFLD and its progression to fibrosis. This dual approach leverages the dietary induction of steatosis and metabolic disturbances with the chemical induction of liver injury and fibrosis [108]. The high-fat diet component closely mimics the dietary habits that contribute to human NAFLD, such as the consumption of calorie-dense, fatty foods [105].

By combining a high-fat diet with CCl_4_, this model effectively replicates the full spectrum of NAFLD, from simple steatosis to severe fibrosis that develops in a short period of time, allowing researchers to study the disease’s entire progression within a single model. However, CCl_4_ is highly toxic and can cause significant morbidity and mortality in animals. The severity of liver damage induced by CCl_4_ can sometimes overshadow the dietary effects, making distinguishing between the effects of the high-fat diet and those of CCl_4_ challenging, particularly when investigating specific mechanisms [108,109].

Moreover, CCl_4_ induces acute liver injury, whereas human NAFLD develops over years or decades. The short-term nature of CCl_4_-induced damage may not fully capture the chronic and progressive aspects of human NAFLD.

A high-fat diet in combination with CCl_4_ was shown to induce all histopathological features of NASH (fatty liver change, inflammation, ballooning of hepatocytes, and fibrosis) followed by increased activities of serum alanine aminotransferase. Multiple administrations of CCl_4_ in obese mice reduced the ratio of reduced glutathione to oxidized glutathione, SOD activity, and mitochondrial DNA copy number, contributing to the development of chronic oxidative stress, increased numbers of apoptotic cells, and increased levels of both TNF-α and TGF-β mRNA. These mechanisms lead to the progression of fatty liver to steatohepatitis and liver fibrosis [109]. A recent study using the same model of NASH-induced fibrosis (HFD+CCl_4_) showed reduced activity of antioxidant enzymes SOD and catalase and reduced GSH content in liver tissue, and liver histopathology confirmed the presence of inflammation, fibrosis, and necrosis [110]. Administration of CCl_4_ to a Western diet supplemented with fructose (WDF) reduced induction time and exacerbated liver fibrosis in NASH mice. In this model, increased steatosis, inflammation, hepatocyte ballooning, bridging, and liver hydroxyproline content were observed [111]. HFD+CCl_4_-induced NASH was investigated in a recent study by Keshavarz et al. [112]. These animals showed a significant increase in body and liver weight compared to the control group after four weeks. Liver fibrosis was confirmed by trichrome staining and was accompanied by a significant increase in proinflammatory IL-6, TNF-α, TLR-2, and TLR-4 and a decrease in anti-inflammatory IL-10. In liver tissue, there were increased markers of liver fibrosis evident as increased TGF-β, α-SMA, TIMP-1, collagen type I, and PDGF mRNA expression.

### Advantages and Limitations of Various Animal Models of Lean NAFLD-Induced Fibrosis

Animal models of NAFLD are indispensable tools for understanding the pathophysiological mechanisms underlying the disease and its progression to fibrosis. The choice of model must be carefully aligned with the specific research objectives and the clinical features of human NAFLD to maximize relevance and translatability. The MCD diet is highly effective in inducing steatosis, inflammation, and fibrosis, rapidly advancing the disease state in a reproducible manner. However, it lacks the metabolic syndrome features typical of human NAFLD, such as obesity and insulin resistance, and results in weight loss, which limits its clinical applicability [76,77,80]. The CDAA diet model also induces significant fibrosis and provides a lean phenotype, useful for studying NAFLD independent of obesity. Nonetheless, it similarly fails to replicate the metabolic syndrome features and involves an artificial dietary deficiency uncommon in human populations, which may impact its translatability [80,81]. The high-fructose diet model closely mimics modern human dietary patterns and induces features of metabolic syndrome, including insulin resistance and dyslipidemia with slow progression from steatosis to fibrosis [82,83]. However, the variability in response and the differences in metabolic responses between rodents and humans pose challenges for direct clinical translation [85]. The HC diet is valuable for studying the effects of lipid metabolism and oxidative stress, leading to severe liver pathology. This model is relevant to human diets high in cholesterol but may not fully exhibit metabolic syndrome features [84,104,105,106,107]. Combining a high-fat diet with CCl_4_ administration creates a model that encompasses a broad range of NAFLD features, from simple steatosis to severe fibrosis, and induces metabolic syndrome. This combination is powerful for studying synergistic effects and rapid fibrosis induction. However, the use of a potent hepatotoxin like CCl_4_ introduces significant toxicity and mortality risks, ethical concerns, and an artificial mechanism of liver injury that diverges from the chronic nature of human NAFLD [108,109,110,111,112] (Table 2).

In clinical significance, translating the findings from these models to human NAFLD requires careful consideration of the inherent differences between model-induced pathologies and human disease mechanisms. Models incorporating features of metabolic syndrome and dietary patterns prevalent in humans, such as high-fructose and high-fat diets, tend to offer higher clinical relevance. However, integrating findings from multiple models may provide a more comprehensive understanding of NAFLD’s multifactorial nature, necessary for the development of effective therapeutic strategies.

Ultimately, selecting the appropriate animal model for NAFLD research should be guided by the specific clinical features and mechanistic pathways of interest, ensuring that the findings are as relevant and translatable to human conditions as possible.

## 8. Therapeutic Strategies and Clinical Implications

One of the most common causes of liver fibrosis is due to viral hepatitis. A study by Facciorusso et al., a 5-year observational cohort study where the efficacy of antiviral therapy was observed, has shown its effects after five years in patients with hepatitis C-induced fibrosis undergoing this type of treatment. The stiffness of the liver reduces significantly after achieving a response. Also, the degree of reduction is greater in the first year after antiviral treatment [113]. It is an important therapeutic strategy for fibrosis caused by viral hepatitis. In terms of NAFLD, combination treatment is likely to be necessary due to its complex pathophysiology and substantial variety of disease phenotypes. The prevention of obesity and a healthy lifestyle in general still remain pivotal to the prevention and treatment of NAFLD and its complications [114]. As a result of scientific research in the last decade, today, we know with certainty that liver fibrosis is a reversible process. Unfortunately, reversion usually happens too slowly for severe complications to occur. Although numerous anti-fibrotic agents have shown success in experimental animal models, their effects in clinical trials are missing. Therefore, there is still no approved therapy for liver fibrosis [115].

Lifestyle modifications, including weight loss, dietary changes, and regular physical activity, are cornerstones of management for both lean and obese NAFLD patients. Weight loss has been shown to improve liver histology, reduce liver fat content, and decrease the risk of disease progression in NAFLD, regardless of body composition. In addition to lifestyle interventions, pharmacotherapy options may be considered for certain patients with NAFLD. Insulin sensitizers such as pioglitazone and GLP-1 receptor agonists have shown promise in improving liver histology and metabolic parameters in patients with NAFLD, although further research is needed to define their role in clinical practice [116]. Lipid-lowering agents, antioxidants, and anti-inflammatory agents are also under investigation as potential treatments for NAFLD and its associated comorbidities [117]. Management strategies for lean NAFLD individuals may involve lifestyle modifications such as weight loss, dietary changes, and regular physical activity, aimed at improving metabolic health and reducing liver fat accumulation. Pharmacotherapy options, including insulin sensitizers, lipid-lowering agents, and anti-inflammatory agents, may also be considered in select cases [115]. Additionally, close monitoring for disease progression and complications, such as screening for liver fibrosis and hepatocellular carcinoma, is essential for optimizing clinical outcomes in lean NAFLD individuals.

## 9. Conclusions

Lean NAFLD represents a distinct clinical phenotype characterized by liver fat accumulation in individuals with a lower BMI and less overall adiposity compared to obese NAFLD individuals. Differences in body composition, metabolic profile, and underlying molecular mechanisms contribute to variations in disease presentation, progression, and management strategies between lean and obese NAFLD individuals. Comparing different animal models of lean NAFLD, the most common mechanisms of liver fibrosis include the overexpression of TGF-β, α-SMA, and collagen type I. Additional factors that contribute to liver fibrogenesis in lean NAFLD models include MMPs, TIMPs, PPARγ, TLR2, and TLR4 and the overactivation of HSCs. In most lean NAFLD models, oxidative stress is a central mechanism triggering fibrosis. The accumulation of ROS and lipid peroxidation products causes cellular damage and activates inflammatory pathways. This oxidative stress is a result of disrupted mitochondrial function and impaired antioxidant defenses. Cell injury causes the upregulation of proinflammatory cytokines such as TNF-α, IL-6, and IL-1β, which are produced by hepatocytes, Kupffer cells, and infiltrating immune cells, contributing to a proinflammatory hepatic environment. This chronic inflammation promotes the activation of HSCs.

In response to liver injury and inflammation, HSCs transdifferentiate into myofibroblast-like cells that produce collagen and other ECM components. This process is driven by TGF-β and other fibrogenic signals such as lipotoxicity. ER stress is a significant contributor to fibrosis in lean NAFLD models. Nutrient deficiencies and lipotoxicity lead to the accumulation of misfolded proteins in the ER, activating the unfolded protein response. Chronic ER stress can trigger apoptosis and further inflammatory signaling, exacerbating liver injury and fibrosis.

Understanding fibrosis development and molecular mechanisms in lean NAFLD models is crucial for addressing the disease in patients who do not exhibit typical metabolic risk factors. These models highlight the importance of oxidative stress, inflammation, ER stress, and disrupted lipid metabolism in driving fibrosis, independent of obesity. This knowledge can aid the development of targeted therapies that address these specific pathways, offering potential treatments for lean NAFLD patients

## Figures and Tables

**Table 1:** Characteristics of lean NAFLD rodent models. MCD, methionine-choline-deficient; CDAA, choline-deficient L-amino-defined high fat diet; CDAHFD, choline deficiency, l-amino acid-defined high-fat diet; HFD, high-fat diet; PPAR-α, peroxisome proliferator-activated receptors; CCl_4_, carbon tetrachloride.

Rodent Model	Obesity	Insulin Resistance	Steatosis	Fibrosis
***Dietary***				
MCD	Weight loss	No	++	+++
CDAA	No	No	++	++
CDAHFD	No	No	+++	++
High-fructose diet	No	Yes	++	No
High-cholesterol diet	No	Yes	++	++
***Genetic***				
PPAR-α -/-	No	No	++	+
CD36-/-	No	Yes	+++	No
***Toxic***				
Tetracycline	No	No	+++	++
CCl_4_	No	No	+++	+++
***Combined model***				
HFD+CCl_4_	Weight gain	No	+++	+++

**Table 2:** Advantages and limitations of different animal models of lean NAFLD.

Animal Model	Advantages	Limitations
**MCD diet**	Effectively induces steatosis, inflammation, and fibrosis. Rapid development of liver pathology. Simple and reproducible model.	Lacks features of metabolic syndrome. Causes weight loss. Dietary composition not representative of typical human diets.
**CDAA diet**	Induces significant liver fibrosis. Consistent and reproducible model. Lean phenotype useful for studying NAFLD independent of obesity.	Lack of metabolic syndrome. Weight loss in animals. Choline deficiency is rare in human diets, limiting relevance.
**High fructose diet**	Induces metabolic syndrome features. Relevant to modern human diets. Progressive liver pathology.	Variable severity and duration required for fibrosis development.
**High cholesterol diet**	Mimics dietary influences on human NAFLD. Induces severe liver pathology. Valuable for studying lipid metabolism. Can be combined with other models.	May not fully exhibit metabolic syndrome features. Artificially high cholesterol levels. Duration and severity of effects can vary.
**High fat diet + CCl_4_**	Relevant to human dietary patterns. Comprehensive disease representation. Rapid and severe fibrosis.	High toxicity and potential for mortality. Increased complexity in experimental design. Shorth-term effects may not capture chronic aspects of human NAFLD.

## Data Availability

Data are contained within the article.
